# Identification of *BMP10* as a Novel Gene Contributing to Dilated Cardiomyopathy

**DOI:** 10.3390/diagnostics13020242

**Published:** 2023-01-09

**Authors:** Jia-Ning Gu, Chen-Xi Yang, Yuan-Yuan Ding, Qi Qiao, Ruo-Min Di, Yu-Min Sun, Jun Wang, Ling Yang, Ying-Jia Xu, Yi-Qing Yang

**Affiliations:** 1 Department of Cardiology, Shanghai Fifth People′s Hospital, Fudan University, Shanghai 200240, China; 2 Shanghai Health Development Research Center, Shanghai Medical Information Center, Shanghai 200031, China; 3 Department of Cardiology, Shanghai Jing’an District Central Hospital, Fudan University, Shanghai 200040, China; 4 Department of Ultrasound, Shanghai Chest Hospital, School of Medicine, Shanghai Jiao Tong University, Shanghai 200030, China; 5 Department of Cardiovascular Research Laboratory, Shanghai Fifth People′s Hospital, Fudan University, Shanghai 200240, China; 6 Department of Central Laboratory, Shanghai Fifth People′s Hospital, Fudan University, Shanghai 200240, China

**Keywords:** dilated cardiomyopathy, molecular genetics, transcriptional regulation, BMP10, reporter gene analysis

## Abstract

Dilated cardiomyopathy (DCM), characterized by left ventricular or biventricular enlargement with systolic dysfunction, is the most common type of cardiac muscle disease. It is a major cause of congestive heart failure and the most frequent indication for heart transplantation. Aggregating evidence has convincingly demonstrated that DCM has an underlying genetic basis, though the genetic defects responsible for DCM in a larger proportion of cases remain elusive, motivating the ongoing research for new DCM-causative genes. In the current investigation, a multigenerational family affected with autosomal-dominant DCM was recruited from the Chinese Han population. By whole-exome sequencing and Sanger sequencing analyses of the DNAs from the family members, a new *BMP10* variation, NM_014482.3:c.166C > T;p.(Gln56*), was discovered and verified to be in co-segregation with the DCM phenotype in the entire family. The heterozygous *BMP10* variant was not detected in 268 healthy volunteers enrolled as control subjects. The functional measurement via dual-luciferase reporter assay revealed that Gln56*-mutant BMP10 lost the ability to transactivate its target genes *NKX2.5* and *TBX20*, two genes that had been causally linked to DCM. The findings strongly indicate *BMP10* as a new gene contributing to DCM in humans and support *BMP10* haploinsufficiency as an alternative pathogenic mechanism underpinning DCM, implying potential implications for the early genetic diagnosis and precision prophylaxis of DCM.

## 1. Introduction

Dilated cardiomyopathy (DCM), which is characteristic of left ventricular or biventricular enlargement with systolic dysfunction, represents the most common form of cardiac muscle disease in humans, affecting up to 1 in 250 persons in the general population [1,2]. The prevalence of DCM is slightly higher in men, with a ratio of female to male ranging from 1:1.3 to 1:1.5 [3]. DCM can result in many adverse clinical consequences, including thromboembolic complications [4,5], cardiac conduction system abnormalities and life-threatening ventricular arrhythmias [6,7,8,9], cardiac dysfunction and congestive heart failure [10,11,12,13,14,15], and even sudden death [16,17,18,19]. In fact, DCM is the third most prevalent etiology of heart failure and the most common indication for heart transplantation [3]. Additionally, the annual incidence of sudden cardiac death in patients with DCM is between 2% and 4%, and in a registry of survivors of aborted sudden cardiac death, DCM underlies etiology in 10%–19% [3]. Merely in the United States, DCM is accountable for approximately 10,000 deaths and 46,000 hospitalizations each year, making it among the top frequent causes of fatalities and an enormous burden on the healthcare system [20]. Hence, DCM is responsible for substantial morbidity and mortality as well as a heavy socioeconomic burden, highlighting the urgent necessity to identify the etiologies underpinning DCM.

The etiologies of DCM are complex and diverse, and both environmental risk factors and inherited defects have been implicated in the development of DCM [1,2,21,22]. Well-known environmental precipitating factors for DCM include viral or bacterial myocarditis, coronary heart disease, autoimmune disorders, tachycardias, neuromuscular disease, diabetes, nutritional deficiency, pregnancy, and exposure to drugs or toxins [3,20,23]. However, there is a growing body of evidence convincingly demonstrating the critical role of genetic determinants in the occurrence of DCM, especially for familial DCM, which is defined when >1 first-degree relative has been diagnosed with DCM or has suffered sudden cardiac death with an age of ≤35 years [1,2,20,21,24]. DCM is inherited in 20–50% of patients and inheritance of DCM is predominantly autosomal dominant, while less common manners of inheritance, including autosomal-recessive inheritance and X-linked recessive inheritance, as well as mitochondrial inheritance, have also been observed in some cases [20,24]. To date, DCM-causative mutations in over 250 genes have been involved in the molecular pathogenesis of DCM, of which the vast majority encode sarcomeric proteins, Z-disk proteins, desmosomal proteins, cytoskeletal proteins, nuclear envelope proteins, sarcolemma proteins, intercalated disc proteins, RNA-binding proteins, mitochondrial proteins, gap junction channel proteins, ion channel proteins, and transcriptional factor proteins, including NKX2.5 and TBX20 [1,2,20,21,24,25,26,27,28,29,30,31,32,33,34,35,36,37,38,39,40,41,42,43,44,45,46,47,48,49,50,51,52,53,54,55,56]. Moreover, genome-wide association studies have revealed a great number of common genetic variations associated with DCM, in addition to a handful of rare new genetic variations related to DCM [57]. However, these known genetic causes are currently estimated to explain 20%–50% of cases, underscoring that additional DCM-causing genes remain to be discovered [1,21].

## 2. Materials and Methods

### 2.1. Research Participants

The current study project involving humans was fulfilled in compliance with the guidelines of the Declaration of Helsinki. The medical ethical committee at the Shanghai Fifth People′s Hospital of Fudan University (Shanghai, China) approved the protocols that were applied to this study (ethical approval code: 2020-011). Each research participant or a legal guardian signed an informed consent form prior to recruitment into the present investigation. For the current investigation, a 4-generation pedigree with a high incidence of an autosomal-dominant pattern of DCM was identified from the Chinese Han ethnicity population (arbitrarily named Family 1), and the family members available were enrolled. A cohort of 268 unrelated healthy volunteers was recruited as the control individuals from the same geographical area in the same population. All research subjects experienced a comprehensive diagnostic evaluation, including a thorough review of personal history, familial history, and medical history; detailed physical examination; transthoracic echocardiogram; and 12-lead electrocardiogram, as well as routine laboratory tests. All the patients affected with DCM also underwent coronary artery angiography and exercise performance tests, but coronary computed tomography angiography, cardiovascular magnetic resonance imaging and endo-myocardial biopsy were conducted only when strongly indicated. A diagnosis of DCM was made as previously described: the presence of a left-ventricular end-diastolic diameter >117% of the predicted value adjusted for the body surface area and age and a left-ventricular ejection fraction of <45% and/or a fractional shortening of <25%, in the absence of abnormal loading conditions, coronary artery disease, exposure to toxins, and other systemic diseases [58,59]. Approximately 2 mL of peripheral venous blood samples from each research participant were collected in tubes containing EDTA anticoagulants. Genomic DNA was isolated from each participant’s blood leucocytes using standard procedures.

### 2.2. Genetic Research

The DNA samples of the affected and unaffected family members from Family 1 with a high incidence of DCM were subjected to whole-exome sequencing (WES), as described elsewhere [60,61,62,63,64]. Briefly, 3 μg of genomic DNA samples from a selected family member were randomly sheared into 100 to 1000 bp fragments using a sonicator (Covaris, Woburn, MA, USA) to construct a whole-exome library, which was ligated to sequencing adapters, enriched for target sequences, and captured with the SureSelect Human All Exon V6 Kit (Agilent Technologies, Santa Clara, CA, USA) and sequenced under a HiSeq 2000 Sequencer (Illumina, San Diego, CA, USA), following the manufacturer’s protocol. The bioinformatics assay of the datasets generated by WES was completed as previously described [60,61,62,63,64]. A Sanger sequencing assay was carried out to verify the candidate genetic variations found by WES analysis. The entire coding region along with splicing donors/acceptors of the gene carrying a validated deleterious variation was sequenced in all the available family members from Family 1 and the 268 control subjects. In addition, such population genetics databases as the Single Nucleotide Polymorphism database (SNP; https://www.ncbi.nlm.nih.gov/, accessed on 12 October 2022) and the Genome Aggregation Database (gnomAD; http://gnomad-sg.org/, accessed on 12 October 2022) were retrieved to check its novelty.

### 2.3. Construction of Recombinant Plasmids

As described elsewhere [60], cDNA was prepared from the total mRNA extracted from the discarded human myocardium, which was derived from a patient undergoing radical surgery for tetralogy of Fallot. The whole open reading frame of wild-type human *BMP10* (accession no. NM_014482.3) was yielded by polymerase chain reaction (PCR) with the Phusion^®^ DNA polymerase (NEB) and a specific pair of primers of 5′-GTGGCTAGCTAAACCTTCCTGGCTTGGCC-3′ (forward) and 5′-CACTCTAGAGCCTCTATTACTGTACACCC-3′ (reverse). The produced full-length *BMP10* cDNA and the pcDNA3.1 plasmid were doubly digested by *Nhe*I and *Xba*I (NEB), purified, and ligated by T_4_ ligase (TaKaRa) to construct the wild-type BMP10-pcDNA3.1 plasmid. The Gln56*-mutant BMP10-pcDNA3.1 plasmid was produced via site-directed mutagenesis employing the QuikChange Lightning Site-Directed Mutagenesis Kit (Agilent) with the complimentary primer pairs of 5′-TTTAACACACTGCTCTAGAGCATGAAGGATG-3′ (forward) and 5′-CATCCTTCATGCTCTAGAGCAGTGTGTTAAA-3′ (backward) and was then confirmed by direct sequencing analysis. The human *NKX2.5* promoter-driven firefly luciferase reporter vector (NKX2.5-luc) and the human *TBX20* promoter-driven firefly luciferase reporter plasmid (TBX20-luc) were constructed as described previously [65]. All the final recombinant constructs were verified by Sanger sequencing analysis.

### 2.4. Cell Transfection with Expression Plasmids and Luciferase Reporter Analysis

The HeLa cells were cultivated in Dulbecco’s modified Eagle’s medium (Invitrogen, Waltham, MA, USA) with 10% fetal bovine serum (Thermo Fisher Scientific, Waltham, MA, USA), 100 IU/mL penicillin (Thermo Fisher Scientific), and 100 μg/mL streptomycin (Thermo Fisher Scientific) in a cell incubator at 37 °C with an atmosphere of 5% CO_2_ and 95% air. The cells were seeded in a 24-well plate at a density of 1 × 10^5^ cells/well and maintained for 24 h prior to transient co-transfection with various amounts of recombinant plasmids utilizing the Lipofectamine 3000 Transfection Reagent (Invitrogen), as per the manufacturer’s instructions. Specifically, the HeLa cells were transfected with 0.4 μg of empty pcDNA3.1, or 0.4 μg of wild-type BMP10-pcDNA3.1, or 0.4 μg of Gln56*-mutant BMP10-pcDNA3.1, or 0.2 μg of empty pcDNA3.1 plus 0.2 μg of wild-type BMP10-pcDNA3.1, or 0.2 μg of wild-type BMP10-pcDNA3.1 plus 0.2 μg of Gln56*-mutant BMP10-pcDNA3.1, in combination with 15 ng of pGL4.75 (Promega, Madison, MA, USA) and 1.2 μg of NKX2.5-luc or TBX20-luc. The pGL4.75 vector (Promega), which expresses renilla luciferase, was used as an internal control plasmid to normalize the transfection efficiency among the experimental groups. The transcriptional activity of a promoter was determined quantitatively by measuring the firefly luciferase activity relative to the renilla luciferase activity with the Dual-Glo^®^ Luciferase Assay System (Promega), according to the manufacturer’s protocol. For each plasmid used, a minimum of three independent transfection experiments were fulfilled, and all reporter analyses were made in triplicate. The results are reported as the means of relative luciferase activities and standard deviations.

### 2.5. Statistics

All the measured data are expressed as the mean ± standard deviation. The statistical significance of differences between the two groups was evaluated by Student’s unpaired *t*-test. When comparisons among three or more groups were conducted, one-way ANOVA with a Tukey–Kramer HSD post hoc test was applied. A 2-tailed *p*-value of less than 0.05 was considered to indicate a significant difference.

## 3. Results

### 3.1. Phenotypic Information of the Studied Family Members Suffering from DCM

**Figure 1:**
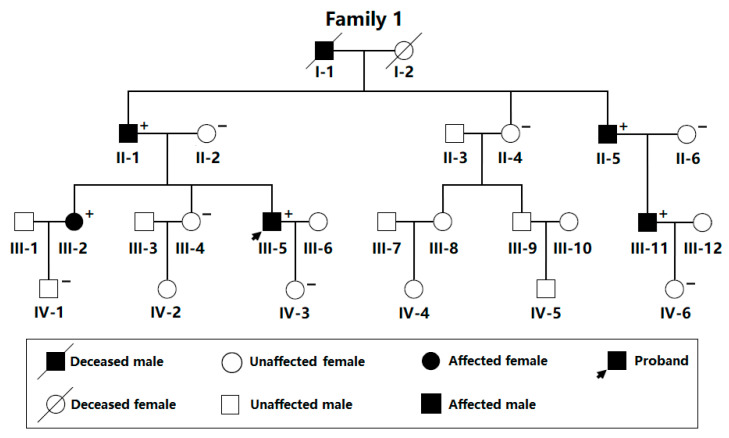
Pedigree of a family affected with dilated cardiomyopathy. Family members are recognized by generations and numbers (Roman–Arabic numbers). A “+” marks a carrier of the detected *BMP10* variation in a heterogeneous status; “–” marks a noncarrier.

In the current study, as shown in Figure 1, a 26-member family suffering from DCM spanning four generations was identified as Family 1 from the Han ethnicity population in China.

**Figure 2:**
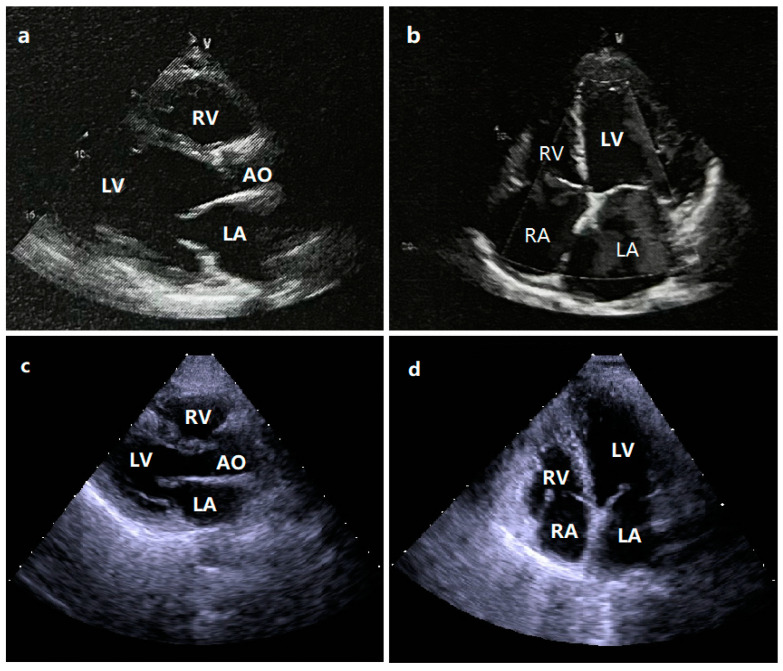
The representative transthoracic two-dimensional echocardiographic images from the proband affected with dilated cardiomyopathy and a healthy individual as a control: (**a**) transthoracic echocardiography findings at the left ventricular longitudinal view from the proband; (**b**) transthoracic echocardiography findings at the apical four-chamber view from the proband; (**c**) transthoracic echocardiography findings at the left ventricular longitudinal view from the control; (**d**) transthoracic echocardiography findings at the apical four-chamber view from the control. The echocardiographic images denote severe left ventricular dilation and systolic dysfunction. LV, left ventricle; RV, right ventricle; LA, left atrium; RA, right atrium; AO, aorta.

In this 26-member family with a high incidence of DCM, there were 24 living members available, including 11 male members and 13 female members. The proband of Family 1 (III-5) was initially diagnosed with DCM at the age of 36 years and received pharmacologic therapies for congestive heart failure, including β-adrenergic receptor antagonist (metoprolol), angiotensin-converting enzyme inhibitor (enalapril), and diuretics (furosemide and spironolactone). His grandfather (I-1) had a past medical history of DCM and died of chronic heart failure resulting from DCM at the age of 62 years. The representative transthoracic two-dimensional echocardiographic images from the proband (III-5) with DCM are shown in Figure 2.

Additionally, in Family 1 (shown in Figure 1), all of the six affected members were diagnosed with DCM based on the findings of a transthoracic echocardiography as well as the cardiologist’s clinical judgment, whereas all the unaffected family members had no history of DCM, with normal echocardiography parameters. No members from Family 1 had structural heart diseases or other known diseases predisposing to DCM, such as rheumatic heart disease, coronary artery disease, viral myocarditis, autoimmune disorders, and arterial hypertension. A genetic assessment of the family suggested that the DCM phenotype was transmitted apparently in an autosomal-dominant mode amongst the family members, with complete penetrance. The baseline clinical characteristic information of the living family members affected with DCM from Family 1 is summarized in Table 1.

### 3.2. Identification of a Novel DCM-Causing Variation in BMP10

A WES was carried out in three DCM-affected pedigree members (II-1, III-5, and III-11 from Family 1) and three unaffected members (II-2, III-6, and III-12 from Family 1), which generated approximately 22 giga bases of sequence data for each member, showing nearly 98% coverage of the human genome (GRCh37/hg19) with approximately 78% mapped to the target region. An average of 16,962 nonsynonymous variations (varying from 15,639 to 18,207) per pedigree member passed filtering in terms of the likely genetic transmission fashions (the recessive inheritance pattern was also considered and included during the screening of candidate genes/variants) of which 10 heterozygous nonsense and missense variations passed filtering by ANNOVAR, with a minor allele frequency being <0.1% and being harbored by all the three DCM-affected members (II-1, III-5, and III-11 from Family 1) who underwent WES, as given in Table 2.

Furthermore, the Sanger sequencing analysis with the *BMP10*-specific primers presented in Table 3 unveiled that merely the nonsense variation of chr2:69,098,325C > T (GRCh37.p13/hg19: NC_000002.11), equal to chr2:68,861,113C > T (GRCh38.p14/hg38: NC_000002.12) or NM_014482.3: c.166C > T;p.(Gln56*), in *BMP10* was confirmed and validated to co-segregate with DCM in the whole family, with complete penetrance, being shared by all affected and none of the unaffected members. Of the other nine missense variations, eight variations were also observed in the unaffected members, while the remaining one variation was absent from one affected member (III-2) in the same family. Hence, these nine genetic variations are unlikely to account for DCM in this family.

**Figure 3:**
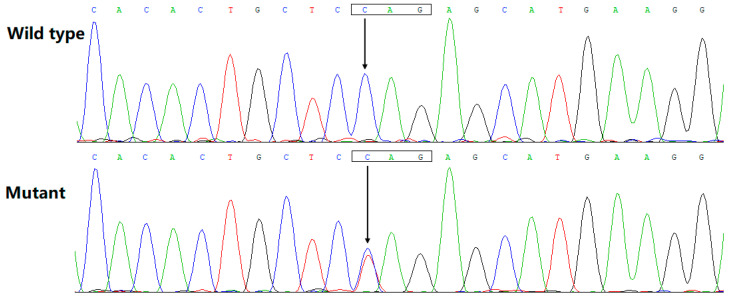
A new *BMP10* variation responsible for dilated cardiomyopathy. The Sanger sequencing chromatograms confirmed the heterogeneous *BMP10* variation (C/T) in the affected proband (mutant) in comparison with homozygous wild-type bases (C/C) in an unaffected individual (wild type). A rectangle delimits a codon. An arrow depicts the position where the variation occurs.

The chromatograms from the Sanger sequencing exhibiting the heterozygous *BMP10* variant (C/T) as well as its wild-type homozygous control (C/C) are illustrated in Figure 3. The discovered *BMP10* variation was neither detected in 536 referential human chromosomes nor retrieved in such databases as SNP (accessed on 19 October 2022) and gnomAD (accessed on 19 October 2022), indicating it as a new *BMP10* variation. This *BMP10* variant, NM_014482.3: c.166C > T;p.(Gln56*), was deposited in a public genetics database called the Leiden Open Variation Database (https://databases.lovd.nl/shared/genes/BMP10, accessed on 4 December 2022), with a variant number of 0000896420 (https://databases.lovd.nl/shared/variants/0000896420) and with an individual number of 00420368 (https://databases.lovd.nl/shared/individuals/00420368, accessed on 4 December 2022).

### 3.3. Inability of Gln56*-Mutant BMP10 to Transactivate NKX2.5

**Figure 4:**
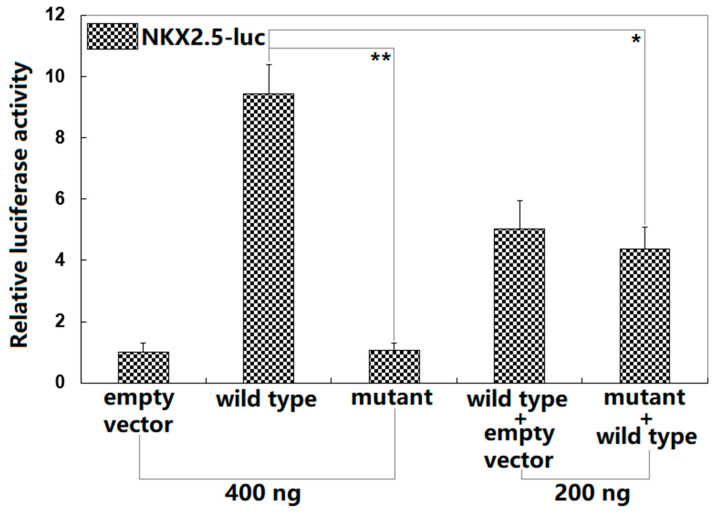
Functional failure of BMP10 caused by the Gln56* variation. The dual-reporter analysis of the transactivation of the *NKX2.5* promoter-driven luciferase expression in cultured HeLa cells by wild-type BMP10 (wild type) or Gln56*-mutant BMP10 (mutant), singly or in combination, revealed that the mutant lost transcription activity. For each expression plasmid used, three independent transfection experiments were carried out in vitro in triplicate. The student’s unpaired *t*-test was employed to compare the two groups. Herein * and ** indicate *p* < 0.0005 and *p* < 0.005, respectively, in comparison with the wild type (400 ng).

As shown in Figure 4, in the cultivated HeLa cells transiently transfected with various expression plasmids, wild-type BMP10 and Gln56*-mutant BMP10 transcriptionally activated the *NKX2.5* promoter by ~9-fold and ~1-fold, respectively (comparison of Gln56*-mutant BMP10 with wild-type BMP10: t = 14.8417; *p* = 0.00012). When Gln56*-mutant BMP10 and wild-type BMP10 were co-transfected, the induced transcriptional activity was ~4-fold (wild-type BMP10 versus wild-type BMP10 plus Gln56*-mutant BMP10: t = 7.45241; *p* = 0.00173). When multiple comparisons were performed, similar statistical results were obtained (F = 144.307, *p* = 1.863 × 10^−7^). Multiple comparisons were made between 0.4 μg of wild-type BMP10-pcDNA3.1 and 0.4 μg of empty plasmid pcDNA3.1 (t = 8.4233, *p* < 0.00001), between 0.4 μg of wild-type BMP10-pcDNA3.1 and 0.4 μg of Gln56*-mutant BMP10-pcDNA3.1 (t = 8.3667, *p* < 0.00001), between 0.4 μg of wild-type BMP10-pcDNA3.1 and 0.2 μg of wild-type BMP10-pcDNA3.1 + 0.2 μg of empty plasmid pcDNA3.1 (t = 4.4, *p* = 0.00010), between 0.4 μg of wild-type BMP10-pcDNA3.1 and 0.2 μg of wild-type BMP10-pcDNA3.1 + 0.2 μg of Gln56*-mutant BMP10-pcDNA3.1 (t = 5.0667, *p* = 0.00003), between 0.4 μg of Gln56*-mutant BMP10-pcDNA3.1 and 0.4 μg of empty plasmid pcDNA3.1 (t = 0.0567, *p* = 0.99997), between 0.4 μg of Gln56*-mutant BMP10-pcDNA3.1 and 0.2 μg of wild-type BMP10-pcDNA3.1 + 0.2 μg of empty plasmid pcDNA3.1 (t = 3.9667, *p* = 0.00025), between 0.4 μg of Gln56*-mutant BMP10-pcDNA3.1 and 0.2 μg of wild-type BMP10-pcDNA3.1 + 0.2 μg of Gln56*-mutant BMP10-pcDNA3.1 (t = 3.3, *p* = 0.00112), between 0.4 μg of empty plasmid pcDNA3.1 and 0.2 μg of wild-type BMP10-pcDNA3.1 + 0.2 μg of empty plasmid pcDNA3.1 (t = 4.0233, *p* = 0.00022), between 0.2 μg of wild-type BMP10-pcDNA3.1 + 0.2 μg of empty plasmid pcDNA3.1 and 0.2 μg of wild-type BMP10-pcDNA3.1 + 0.2 μg of Gln56*-mutant BMP10-pcDNA3.1 (t = 0.6667, *p* = 0.75691), and between 0.4 μg of empty plasmid pcDNA3.1 and 0.2 μg of wild-type BMP10-pcDNA3.1 + 0.2 μg of Gln56*-mutant BMP10-pcDNA3.1 (t = 3.3567, *p* = 0.00098). Here, the Gln56*-mutant BMP10 + wild-type BMP10 group was conducted in vitro to mimic the pathogenic status of the DCM patients harboring the heterozygous *BMP10* mutation; the wild-type BMP10 group and the wild-type BMP10 + Gln56*-mutant BMP10 group were performed to explore the potential dominant-negative effect of Gln56*-mutant BMP10 on wild-type BMP10, and the results suggested no dominant-negative effect of Gln56*-mutant BMP10 on wild-type BMP10.

### 3.4. Failure of Gln56*-Mutant BMP10 to Transactivate TBX20

**Figure 5:**
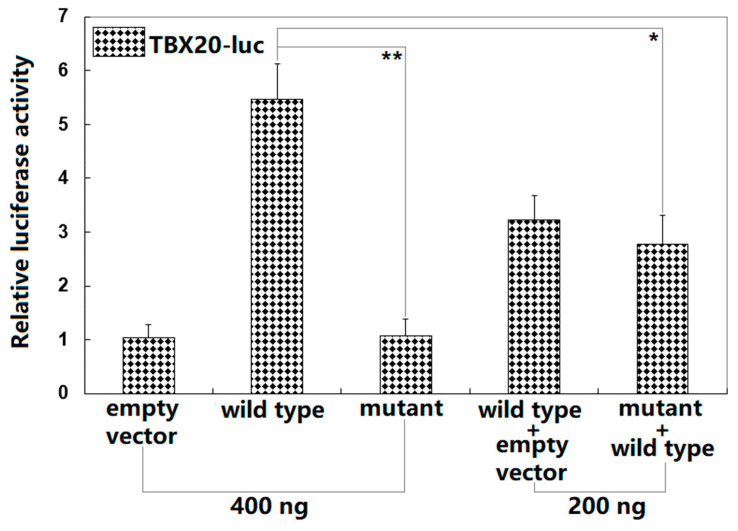
Functional loss of BMP10 resulted from the Gln56* variation. The reporter assay of the transcriptional activation of the *TBX20* promoter-driven luciferase expression in cultivated HeLa cells by wild-type BMP10 or Gln56*-mutant BMP10 (Gln56*), alone or together, unveiled that the Gln56* variant had no transcriptional activity. For each expression plasmid utilized, three independent transfection experiments in vitro were conducted in triplicate. The student’s unpaired *t*-test was applied to make the comparison between the two groups. Herein * and ** indicate *p* < 0.001 and *p* < 0.01, respectively, in comparison with wild-type BMP10 (400 ng).

As displayed in Figure 5, in cultured HeLa cells overexpressing interest proteins, wild-type BMP10 and Gln56*-mutant BMP10 transactivated the *TBX20* promoter by ~5-fold and ~1-fold, respectively (comparison of Gln56*-mutant BMP10 with wild-type BMP10: t = 10.446; *p* = 0.00047). When Gln56*-mutant BMP10 and wild-type BMP10 were transfected in combination, the elicited transcriptional activity was ~3-fold (wild-type BMP10 versus wild-type BMP10 plus Gln56*-mutant BMP10: t = 5.41204; *p* = 0.00565). When multiple comparisons were made, similar statistical results were achieved (F = 40.164, *p* = 2.327 × 10^−6^). Multiple comparisons were made between 0.4 μg of wild-type BMP10-pcDNA3.1 and 0.4 μg of empty plasmid pcDNA3.1 (t = 4.4333, *p* < 0.00001), between 0.4 μg of wild-type BMP10-pcDNA3.1 and 0.4 μg of Gln56*-mutant BMP10-pcDNA3.1 (t = 4.4, *p* < 0.00001), between 0.4 μg of wild-type BMP10-pcDNA3.1 and 0.2 μg of wild-type BMP10-pcDNA3.1 + 0.2 μg of empty plasmid pcDNA3.1 (t = 2.2333, *p* = 0.00128), between 0.4 μg of wild-type BMP10-pcDNA3.1 and 0.2 μg of wild-type BMP10-pcDNA3.1 + 0.2 μg of Gln56*-mutant BMP10-pcDNA3.1 (t = 2.7, *p* = 0.00028), between 0.4 μg of Gln56*-mutant BMP10-pcDNA3.1 and 0.4 μg of empty plasmid pcDNA3.1 (t = 0.0333, *p* = 0.99998), between 0.4 μg of Gln56*-mutant BMP10-pcDNA3.1 and 0.2 μg of wild-type BMP10-pcDNA3.1 + 0.2 μg of empty plasmid pcDNA3.1 (t = 2.1667, *p* = 0.00161), between 0.4 μg of Gln56*-mutant BMP10-pcDNA3.1 and 0.2 μg of wild-type BMP10-pcDNA3.1 + 0.2 μg of Gln56*-mutant BMP10-pcDNA3.1 (t = 1.7, *p* = 0.00905), between 0.4 μg of empty plasmid pcDNA3.1 and 0.2 μg of wild-type BMP10-pcDNA3.1 + 0.2 μg of empty plasmid pcDNA3.1 (t = 2.2, *p* = 0.00144), between 0.2 μg of wild-type BMP10-pcDNA3.1 + 0.2 μg of empty plasmid pcDNA3.1 and 0.2 μg of wild-type BMP10-pcDNA3.1 + 0.2 μg of Gln56*-mutant BMP10-pcDNA3.1 (t = 0.4667, *p* = 0.74671), and between 0.4 μg of empty plasmid pcDNA3.1 and 0.2 μg of wild-type BMP10-pcDNA3.1 + 0.2 μg of Gln56*-mutant BMP10-pcDNA3.1 (t = 1.7333, *p* = 0.00796).

## 4. Discussion

For the present research, a four-generation pedigree suffering from DCM with an autosomal-dominant mode of inheritance was recruited from the Chinese Han ethnicity population. By WES and bioinformatical analysis in the pedigree members, a heterozygous *BMP10* variation, namely, NM_014482.3: c.166C > T;p.(Gln56*), was found and validated through a Sanger sequencing assay to be in co-segregation with the DCM phenotype in the whole pedigree. This *BMP10* variation was neither detected in 536 referential human chromosomes nor released from the SNP and gnomAD databases. The quantitative reporter assays demonstrated that Gln56*-mutant BMP10 lost the ability to transactivate the promoters of *NKX2.5* and *TBX20*, two DCM-causative genes [66,67,68,69,70,71,72,73,74,75]. These results strongly support the notion that genetically defective *BMP10* contributes to the occurrence of DCM in humans. Nevertheless, if the patients’ in vivo samples such as lymphoid cells could be obtained and explored with functional analysis, more solid evidence supporting the pathogenic effect of the mutation would be acquired.

In humans, *BMP10* mapped on chromosome 2p13.3, coding for a cardiac peptide growth factor with 424 amino acids, a member of the bone morphogenetic protein (BMP) family of ligands, belonging to the transforming growth factor beta (TGF-β) superfamily that critically regulates cardiovascular growth, development, and maturation [76]. The BMP family of members regulates a wide spectrum of developmental events and physiological functions throughout evolution in various species from insects to mammals [76]. Although all members from the BMP family possess similar protein structures, each member has a distinct pattern of tissue distribution and unique functional effect [77,78]. Presently, at least six BMP members were found to be expressed in the heart, including BMP10, BMP7, BMP6, BMP5, BMP4, and BMP2, of which only BMP10 shows a specific and enriched expression in the heart [79,80]. BMP10 is amply expressed in the hearts of chicks, mice and humans [76]. In developing hearts, BMP10 is more enriched in the trabecular myocardium, whereas in adult hearts, it is abundantly expressed in the right atrium, though the BMP10 protein can be detected throughout the heart [80]. Of note, in adult ventricular cardiomyocytes, the expression of BMP10 is elevated in response to hypertension, indicating that BMP10 is involved in postnatal cardiac physiology [76,81]. BMP10 has been demonstrated to elicit intracellular signaling via the receptor complex of activin receptor-like kinase 1 (ALK1) with activin receptor type 2A or morphogenetic protein receptor type II [82,83]. Recently, BMP10 has been validated to activate two important intracellular signaling pathways, the SMAD-mediated canonical pathway and the STAT3-mediated noncanonical pathway [76], and induce the expression of multiple target genes key to embryonic cardiovascular morphogenesis and postnatal cardiovascular structural remodeling through the SMAD-binding sites in the promoters of target genes, including *TBX20*, *NKX2.5,* and *MEF2C* [80,84,85], three genes that have been causally linked to DCM [66,67,68,69,70,71,72,73,74,75,86]. In the current investigation, the identified Gln56* mutation was anticipated to generate a truncated BMP10 protein losing most functional domains, and a functional assay unveiled that Gln56*-mutant BMP10 did not induce the expression of the genes *TBX20* and *NKX2.5*. These findings indicate that *BMP10* haploinsufficiency is an alternative molecular mechanism underlying DCM in humans.

It may be ascribed to abnormal cardiac development and structural reconstruction that *BMP10* mutation predisposes to DCM. In mice, homozygous deletion of *Bmp10* led to embryonic lethality due to the fact of profound defects in cardiac development and severely impaired heart function, though heterozygous *Bmp10*-deficient mice were viable and fertile [80]. In the *Bmp10*-knockout murine embryos, cardiac morphogenesis appeared to be arrested with profound hypoplastic ventricular walls and lack of ventricular trabeculae, and the development of endocardial cushions was anomalous in both atrioventricular canal and outflow tract and was halted at the acellular stage [80]. Further studies suggested that BMP10 had a growth-promoting activity for embryonic cardiomyocytes, and a markedly reduced proliferation of *Bmp10*-null cardiomyocytes was accountable for the severely hypoplastic and thinned ventricular wall as well as little ventricular trabeculation [80]. Furthermore, although the mice with a single knockout of Bmp9 or Bmp10 displayed no obvious phenotypes, the mice with a double knockout of Bmp9 and Bmp10 resulted in vascular defects and high-output heart failure as well as pulmonary hemosiderosis [87]. In addition, in the murine embryos with the genetic replacement of *Bmp10* with *Bmp9*, the hearts were hypoplastic with significantly thinner ventricular walls, overtly abnormal in the gross morphology with enlarged size, altered shape, pronounced ventricular septal defects, reduced cell cycle activity, and marked pericardial edema, reminiscent of the phenotypes of the *Bmp10*-null embryos [82]. In addition, the MYOCD/BMP10 signaling pathway has been discovered to be required for proper cardiac growth, chamber maturation, and embryonic survival, and *Myocd*-null embryos showed myocardial hypoplasia, defective atrial and ventricular chamber maturation, heart failure, and embryonic lethality, which were caused in part by a block in the BMP10 signaling [88]. In contrast, in hypertensive rats, the expression of BMP10 was increased in the hypertrophied ventricles [81]. Moreover, overexpression of BMP10 in the adult hearts of conditional *Bmp10* transgenic mice or intraperitoneal administration of BMP10 was observed to effectively protect hearts from injury and dysfunction, and the BMP10′s cardioprotective function could be due to the fact of its dual activation of the SMAD- and STAT3-mediated signaling pathways, promoting myocardial survival and inhibiting cardiac fibrosis. [76]. In humans, a rare *BMP10* variation, c.977C > T; p.(Thr326Ile), was discovered to be significantly associated with hypertensive dilated cardiomyopathy [81]. Functional analysis revealed that the Thr326Ile-mutant BMP10 showed decreased binding to Titin-cap at stretch-sensing Z discs of cardiomyocytes and increased extracellular secretion, and conditioned medium from the cells transfected with either wild-type or Thr326Ile-mutant BMP10 stimulated hypertrophy in rat neonatal cardiomyocytes [81]. Taken collectively, these data suggest that genetically compromised *BMP10* predisposes to DCM in humans.

Previous research has substantiated that a premature translation termination codon may lead to the degradation of mRNA in distinct types of organisms and cell lines via a mechanism called nonsense-mediated mRNA decay (NMD), a translation-dependent, multistep process that monitors and degrades faulty or irregular mRNAs [89]. In the current investigation, the nonsense mutation in BMP10 generated a premature translation termination codon; hence, the mutant BMP10 mRNAs were likely to undergo NMD, though not all nonsense mutations triggered it [90]. Presently, we could not exclude NMD in the BMP10 mutation carriers mainly owing to the unavailability of their cardiac tissue samples, where the mutant BMP10 protein might be expressed. Even though the mutant BMP10 mRNAs underwent NMD, the overall quantity of BMP10 mRNA would be decreased by half, resulting in haploinsufficiency, which was consistent with our functional data. Notably, downstream intron or pre-mRNA splicing, which is required for the deposition of a multiprotein complex, named the exon–junction complex, approximately 20–24 nucleotides upstream of each exon–exon junction, is indispensable for the degradation of mRNAs containing a premature translation termination codon through the mechanism of NMD. Therefore, NMD could not ensue in the condition of cDNA constructs [89].

## 5. Conclusions

The current investigation strongly indicates *BMP10* as a novel gene contributing to DCM in humans, implying potential implications for antenatal prophylaxis, prognostic risk assessment, and even individualized treatment of DCM in a subset of patients.

## Figures and Tables

**Table 1:** Baseline clinical characteristic information of the living pedigree members available from Family 1.

Individual (Family 1)	Sex	Age (Years)	Phenotype	LVESD (mm)	LVEDD (mm)	LVEF (%)	LVFS (%)
II-1	Male	67	DCM	70	79	25	12
II-2	Female	66	Normal	30	50	69	39
II-4	Female	65	Normal	30	47	66	36
II-5	Male	61	DCM	53	65	37	18
II-6	Female	59	Normal	29	44	62	33
III-1	Male	43	Normal	28	47	71	40
III-2	Female	41	DCM	44	56	44	22
III-4	Female	39	Normal	26	43	69	38
III-5	Male	37	DCM	64	74	29	15
III-6	Female	38	Normal	27	43	68	37
III-11	Male	35	DCM	41	51	40	20
III-12	Female	36	Normal	30	48	62	31
IV-1	Male	18	Normal	28	44	67	35
IV-3	Female	14	Normal	26	41	66	37
IV-6	Female	12	Normal	26	39	64	36

DCM, dilated cardiomyopathy; LVESD, left ventricular end-systolic diameter; LVEDD, left ventricular end-diastolic diameter; LVEF, left ventricular ejection fraction; LVFS, left ventricular fractional shortening.

**Table 2:** Nonsynonymous candidate gene variations for dilated cardiomyopathy discovered by whole-exome sequencing analysis.

Chr	Position (hg19)	Ref	Alt	Gene	Variation
1	74,648,398	C	A	LRRIQ3	NM_001105659.2: c.397C > A;p.(Pro133Thr)
2	69,098,325	C	T	BMP10	NM_014482.3: c.166C > T;p.(Gln56*)
3	167,039,906	A	G	ZBBX	NM_001199201.2: c.982A > G;p.(Arg328Gly)
5	158,621,821	A	T	RNF145	NM_001199380.2: c.286A > T;p.(Ser96Cys)
7	99,709,371	A	G	TAF6	NM_005641.4: c.880A > G;p.(Thr294Ala)
8	87,497,109	C	T	RMDN1	NM_016033.3: c.577C > T;p.(His193Tyr)
11	11,976,686	G	A	USP47	NM_017944.4: c.3664G > A;p.(Asp1222Asn)
15	89,698,709	A	T	ABHD2	NM_007011.8: c.482A > T;p.(Asn161Ile)
18	8,387,195	G	T	PTPRM	NM_001105244.2: c.4170G > T;p.(Glu1390Asp)
20	40,033,543	T	C	CHD6	NM_032221.5: c.7838T > C;p.(Leu2613Pro)

Alt, alteration; Chr, chromosome; Ref, reference.

**Table 3:** Primer pairs to amplify the coding sequences and splicing sites of the *BMP10* gene.

Coding Exon	Forward Primer (5′→3′)	Reverse Primer (5’→3´)	Amplicon Size (bp)
1	CACTTAGAGCCCAGGGAAGC	CCCTTACCTATATCATTCCCATGC	481
2 (a)	GCATCTGTTTTTCCCTGAGACC	TATCCAGGCCCAAGTTGTCC	590
2 (b)	AAGCAGTGACAAGGAGAGGAAGG	GCAGCAAGCCTCTATTACTGTACACC	600

## Data Availability

The data supporting the discovery of the current research are available upon reasonable request.
